# Integrating immersive experience into hybrid education: a case study in fintech experimental education

**DOI:** 10.1038/s41598-023-50259-1

**Published:** 2023-12-20

**Authors:** Tiande Xie, Xiaoyan Wang, Javier Cifuentes-Faura, Yongkang Xing

**Affiliations:** 1 Center of Experimental Teaching, Guangdong University of Finance, Guangzhou, 510521 China; 2 Faculty of Economics and Business, University of Murcia, Murcia, 30003 Spain; 3 Institute of Artificial Intelligence, De Montfort University, Leicester, LE1 9BH UK

**Keywords:** Information technology, Psychology, Human behaviour

## Abstract

The fintech (financial technology) employment market presents significant barriers to entry, including rigorous selection criteria based on factors such as profitability, risk, security, and confidentiality, which limit students’ opportunities to enter the industry. The negative impact of the COVID-19 pandemic has further exacerbated the challenges faced by the fintech employment market in China. Consequently, it is crucial to assist fintech students in enhancing their professional abilities to navigate the job market successfully. Virtual reality is gaining increasing importance in educational fields due to its immersive virtual simulation capabilities. In this study, a hybrid fintech course was designed based on the principles of outcome-based education theory and the flipped classroom model. The project objective was to provide students with virtual training and project-based assessments that facilitate a seamless transition from theoretical knowledge to practical application in the industry. To evaluate the effectiveness of hybrid education, a comparative user experiment was conducted involving 60 participants (students) from Guangdong University of Finance. The study used median data, average data, and the entropy weight method for analysis. The results indicated that hybrid education has a positive impact on individual-level critical thinking, communication, and teamwork skills. We believe that our study can provide critical data references for global online/hybrid education researchers and contribute to the education field.

## Introduction

### Background

As a cutting-edge digital technology, virtual reality (VR) employs head-mounted devices, controllers, or hand recognition to enable interactive experiences within virtual worlds^1^. In recent years, the scope of VR technology has expanded beyond entertainment and has witnessed rapid advancements in various sectors, including media, education, and health care^2^. Through immersive and interactive experiences, VR can bring forth the intricate and often elusive aspects of virtual simulation to users/learners.

The COVID-19 pandemic has profoundly impacted the economic and employment market and global universities, compelling them to transition from traditional on-site teaching to hybrid/online education^3^. The COVID-19 pandemic has also created a cause for rapid innovation in changing education modes to digital at global higher education institutions^4^. Hybrid/online education can guide students to efficiently use online resources to help them expand their career horizons. Additionally, online teaching can avoid geological and time restrictions, while the virtual training platform can enhance practical skills. Given these distinctive attributes of hybrid/online education, online/hybrid education can reduce the unequal quality of education due to geological restrictions and can promote sustainable activities that promote the United Nations Sustainable Development Goals (SDGs)^4^.

Although various online study platforms have been developed during the COVID-19 pandemic, these platforms contain various issues. Previous studies have indicated that some of these platforms lack a real-time feedback module from teachers and have limited interactive communication functions^5,6^. Moreover, specific courses require experimental sections, which general education platforms cannot adequately accommodate. In addition, the connection quality is another significant issue^7^. As such, an unsuitable platform may harm the learning process and lead to reduced efficiency.

Considering the above issues, it is crucial to analyze an appropriate online education solution that aligns with the criteria for experiments in higher education. In Luo’s research (Luo, 2021) , the integration of VR technology showed remarkable potential in enhancing the learning experience^8^. The abovementioned study conducted an extensive background analysis focusing on the utilization of VR projects in higher education, specifically during recent years. In addition, Marks designed a VR laboratory to track the students’ activity over five semesters. The data showed that 71.5% of students can enhance their learning outcomes through VR experimental education^9^. Our comprehensive background study highlights the various benefits of incorporating VR in education, such as creating immersive environments with realistic graphics, interactive elements that augment the learning experience, real-time feedback, and adaptability to incorporate diverse learning criteria^8–11^.

While VR’s interactive and immersive experience can potentially enhance the learning experience in experimental education, many existing VR projects have yet to offer a suitable solution for seamlessly integrating VR content into traditional courses. This may lead to connecting issues between VR projects and the primary curriculum. As the COVID-19 pandemic gradually subsides, higher education institutions are transitioning back to on-site teaching^12^. However, hybrid education continues to evolve and thus requires further development. Therefore, it is essential to explore how the current study can effectively blend VR interactive sessions into on-site teaching and make meaningful contributions to the future of digital education.

### Research scope

To refine the research scope, it is crucial to determine which undergraduate degree programs would benefit from immersive experiments incorporated into their courses.

It is crucial for students who are pursuing degrees in finance and economics to acquire practical experience and internships in financial institutions/companies as part of their comprehensive education^13^. However, due to profitability, risk, security, and confidentiality considerations, most financial institutions/companies in China carefully select only a limited number of internship candidates. Additionally, the COVID-19 pandemic/political impact has damaged the employment market, especially in China^14,15^. As a result, students’ opportunities to engage in internships and gain hands-on experience in financial risk management have been limited, leading to a lack of practical understanding and experience in the systematic processes of the financial industry. While there is significant interest from Chinese educational experts regarding the potential of VR in fintech education, previous studies have discovered that there are limited VR fintech education programs available, and most of them are not openly accessible and lack convincing user test data in China^16^.

Given the aforementioned challenges, it is imperative to construct a suitable hybrid course that incorporates immersive experiments. Such a course should aim to nurture the development of individual financial professional abilities by providing virtual training that bridges the gap between theory and real-world practice. By doing so, this course would have the potential to not only contribute to regional social and economic development but also enhance students’ employment ability and entrepreneurial prospects^17^.

### Research objective

Consequently, the primary objective of this study is to design a financial risk management course in a hybrid mode to enhance students’ professional skills and facilitate their smooth transition into the employment market. The subsequent sections further detail the following research characteristics:

- The study identifies and reviews various theories and proposes a conceptual framework to enhance learning efficiency.
- The study designs a suitable financial management hybrid course and virtual training platform.
- The study conducts a comparison experiment to measure the effectiveness of the course.

## Related work

This study aimed to design a conceptual path to enhance the study experience for a hybrid fintech course with VR technology. Thus, it is necessary to discuss the existing interactive theories and projects available in VR education before pursuing design work. By analyzing these cases, it is possible to devise effective strategies to address them and ensure a high-quality learning experience.

### Concept design (hybrid education)

Outcome-based education (OBE) is an educational theory that sets specific goals (outcomes) for each part of an educational system. The ultimate aim is for every student to achieve these goals by the end of their educational journey^18^. OBE organizes the entire educational system to focus on what learners need to accomplish successfully. When developing curricula and defining outcomes, this approach emphasizes a range of skills, including life skills, basic skills, professional and vocational skills, intellectual skills, and interpersonal and personal skills^18^. OBE theory has been effectively implemented in higher education globally, as evidenced by successful studies^19–21^. Given the course objective of enhancing practical abilities and leading to successful career outcomes, OBE theory aligns well with our research goals and objectives.

The flipped classroom model is a teaching approach that utilizes classroom time for higher-level learning activities, such as discussion, experimentation, and group activities, while using out-of-class time for self-directed learning^22,23^. With digital education continuing to grow, the flipped classroom model has become increasingly popular in hybrid education, especially during the COVID-19 pandemic^24,25^. The flipped classroom model involves students acquiring knowledge before class, actively practicing and applying concepts during classroom interactions with peers and teachers, and, subsequently, reflecting on received feedback to enhance their learning^26^. This process can help students better understand and apply knowledge during their studies and improve their learning efficiency.

In addition to the abovementioned theories, the current study must also focus on the stakeholders. Stakeholders play a crucial role in shaping the structure and content of hybrid courses. Stakeholders include financial industry experts, academic teachers, the VR technology team, and students. First, financial industry experts can utilize their industrial experiences to ensure that the course aligns with industry needs, equipping students with suitable practical skills. Second, teachers should ensure that the course maintains academic rigor and relevance in finance and risk management, taking into account experts’ suggestions. Third, the VR technology team should offer the latest tools and platforms that enhance the learning experience. Last, current and prospective students are essential stakeholders. Surveys, focus groups, and feedback mechanisms should be conducted to help tailor the course to meet their career needs and expectations.

**Figure 1:**
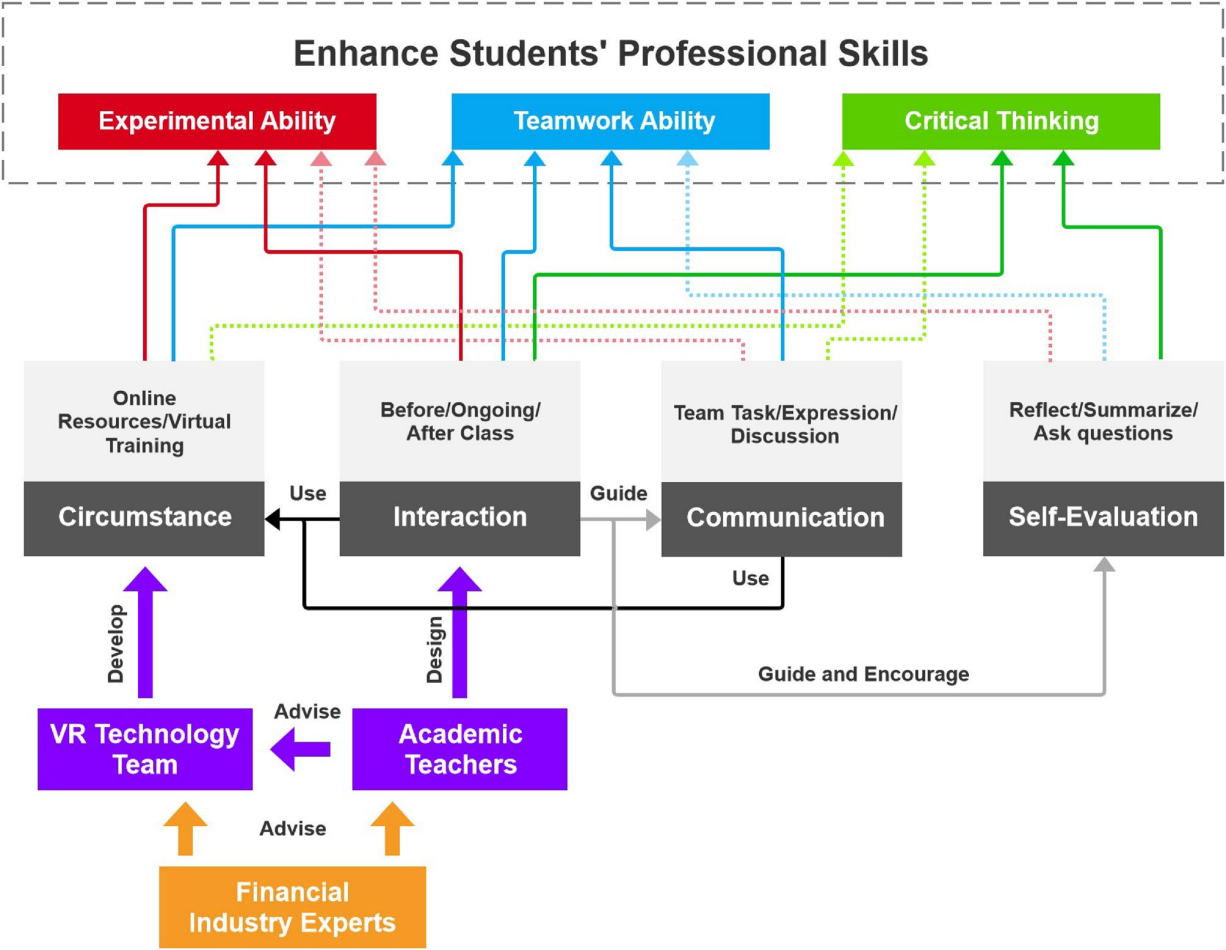
Concept framework based on OBE theory.

Figure 1 illustrates the conceptual design. Drawing on the principles of OBE theory and stakeholders, our first step is to analyze the study objectives for the hybrid course. The primary goal of this study is to enhance individual-level (student-level) professional abilities. Other stakeholders should focus on finding the appropriate means to achieve this goal. Professional abilities encompass not only basic practical skills but also the capacity to communicate effectively with others and apply critical thinking logic, which can benefit their long-term careers. Thus, the current study categorizes these abilities into three key aspects: experimental ability, teamwork ability, and critical thinking. By utilizing the OBE backward design approach, this study ensures that all forms and levels of outcomes across the curriculum are systematically and intentionally aligned and interconnected^27^. This approach allows us to generate a curriculum design and teaching schedule that aligns with our objectives.

The urgent issue to address is how to achieve our objectives effectively. The course should incorporate key elements that contribute to their attainment. Chen identified ten essential elements for designing hybrid courses, namely, creating immersive scenarios, disseminating knowledge, presenting and analyzing problems, introducing key concepts, designing intuitive interfaces and logical structures, facilitating self-evaluation and peer assessment, providing teacher feedback, and encouraging extension and innovation^28^. Similarly, Guanyu emphasized the significance of fostering discussion, notetaking, organizing knowledge in a structured manner, and enabling self-evaluation in the context of smart education^29^.

These previous studies underscored the critical aspects of hybrid courses, which aim to effectively not only train students but also yield favorable outcomes. First, it is necessary to create a smooth online learning environment for disseminating knowledge. Second, academic teachers should carefully arrange teaching sessions with a suitable schedule and efficiently utilize online platforms. These platforms can help students enhance their teamwork efficiency. Third, teachers should play a key role in guiding the students on how to proceed with the project. The online platform should provide efficient services to facilitate collaborative work. Last, the evaluation of personal contributions is also important to provide critical feedback that encourages students to progress. Based on these insights, the current study can conclude that four key aspects are crucial in achieving our objectives: circumstance, interaction, communication and self-evaluation.

**Circumstance:** The course provides a hybrid environment that includes on-site teaching and a VR online platform developed by a VR technology team. Academic teachers and financial industry experts advise the team on how to design the content for each module. The platform also contains abundant digital reading material that can help to improve the quality of online education^30^. The platform can offer online experimental resources for home-based learning, which is especially important in the current situation where many students can attend to experiments after class.

**Interaction:** Teachers need to design teaching schedules with various education interactions. Based on the flipped classroom model, these schedules should focus on times before, during, and after class^26^. Before the class, students should follow the teachers’ instructions to prepare for the session by reviewing the needed materials. During class, teachers should encourage students to actively participate by asking questions and sharing their individual opinions^31^. After class, students should engage in teamwork to validate the hypotheses and theories discussed. To ensure that students receive full support and improve their critical thinking in assessments, teachers should provide tutoring and feedback to help them succeed in the whole process. Additionally, teachers should discuss the experimental content with relevant experts.

**Communication:** Guidelines and rules for teamwork-based projects need to be designed by teachers. Students must follow instructions to clarify the tasks and responsibilities of each team member. Each student should upload their weekly reports to the online platform. Teachers should monitor the reports to ensure that each student has the opportunity to share and express themselves in group discussions. Teamwork training can help students learn how to communicate effectively with colleagues, which can benefit their learning efficiency and future careers^32–34^.

**Self-Evaluation:** Although teachers play a crucial role in determining students’ final scores, individual self-evaluations are necessary. Students need to have a clear understanding of their learning process and the quality of their work. Self-evaluation significantly affects the level of self-directed learning readiness^35^. Students can submit their self-evaluation form online as a progress report to help them reflect and summarize their learning experience and even ask questions. This approach will help them to understand their contributions better and enable the teacher to provide personalized academic support tailored to their specific needs.

### Concept design (VR platform)

To effectively leverage finance knowledge and provide immersive experiences, it is crucial to explore the transformation of finance concepts into a VR platform. Our research has uncovered several theoretical studies based on game typologies, including Bartle’s taxonomy, Keirsey’s personality type theory, and Chris Bateman’s DGD1 model^36,37^. By considering these theoretical frameworks, the current study can select the most appropriate platform interaction type that aligns with the specific requirements of financial risk management content in finance education.

First, the study aims to design the overall exploration process with entertaining experiences and educational knowledge. Danilicheva introduced the concept of virtual storytelling (VST) to create immersive VR educational experiences based on Bartle’s taxonomy^38^. VST emphasizes interactive storytelling, narratives, and dramatic elements, enabling users to actively participate in the system’s story^38^. By engaging with the content through a narrative process, users can shape and influence the storyline^38^. To enhance the storytelling aspect, the system should include a diverse range of characters, encompassing both players (users) and nonplayer characters (NPCs) (Danilicheva, 2009). Storytelling can be approached through two main modes, namely, plot-based and character-based storytelling^39^. Plot-based storytelling requires users to follow a predefined storyline, while character-based storytelling allows for more exploration in an open sandbox environment^39^. In our research, the study will utilize a plot-based storytelling approach to assist students in fully understanding financial experiments during exploration. Since users may not have an extensive amount of time to spend in the system as they would in an open sandbox video game, a plot-based approach aligns with our system’s storytelling mode.

Second, the research should aim to strike a balance between entertainment and education within the system. While VR educational systems share similarities with video games in terms of graphics technology, development processes, and interactive devices, balancing the elements of entertainment and education poses a significant challenge. Thus, it is crucial for the system to prioritize educational elements over entertainment, ensuring that the educational aspect takes precedence.

The current research focuses on designing an overall storyline centered around bank financial risk management, aiming to create an educational experience. The storyline begins with a case study provided by a bank, which serves as the foundation for an interactive bank financial risk management narrative. Within the storyline, students assume the role of a bank staff member and navigate through a commercial bank with a ‘head bank-branch’ structure that is facing liquidity risk caused by an imbalanced asset-liability structure and low liquidity matching rate. Throughout the narrative, students encounter various professional sections, including risk events, management processes, and work scenarios. This immersive experience enables them to acquire relevant financial knowledge and enhance their practical skills, contributing to their future careers.

## Course overview

The financial risk management course is centered around the business and operation of commercial banks, with a focus on risk management, which is considered the core competitiveness of commercial banks. However, there is a shortage of financial risk management experts in China (Mainland and Hong Kong), which is a concern for commercial banks^40,41^. Graduates are expected to possess a comprehensive understanding of financial business, a mastery of financial risk management knowledge, strong financial risk awareness and risk avoidance management skills, and proficiency in financial risk control practice. To address these requirements, the course is designed to incorporate risk management scenarios that integrate production, which can improve students’ practical ability in the financial business.

The course is designed to present a scenario in which a commercial bank with a ‘head bank-branch’ structure is faced with liquidity risk due to an imbalanced asset-liability structure and low liquidity matching rate. The course adopts a hybrid education mode, which includes both on-site lecture teaching and online experiments. Additionally, an online virtual training system is provided to guide students in exploring the simulated bank (accessed on 30 September 2021). The system was developed by the Center of Experimental Teaching, Guangdong University of Finance (our research team’s institute).

**Figure 2:**
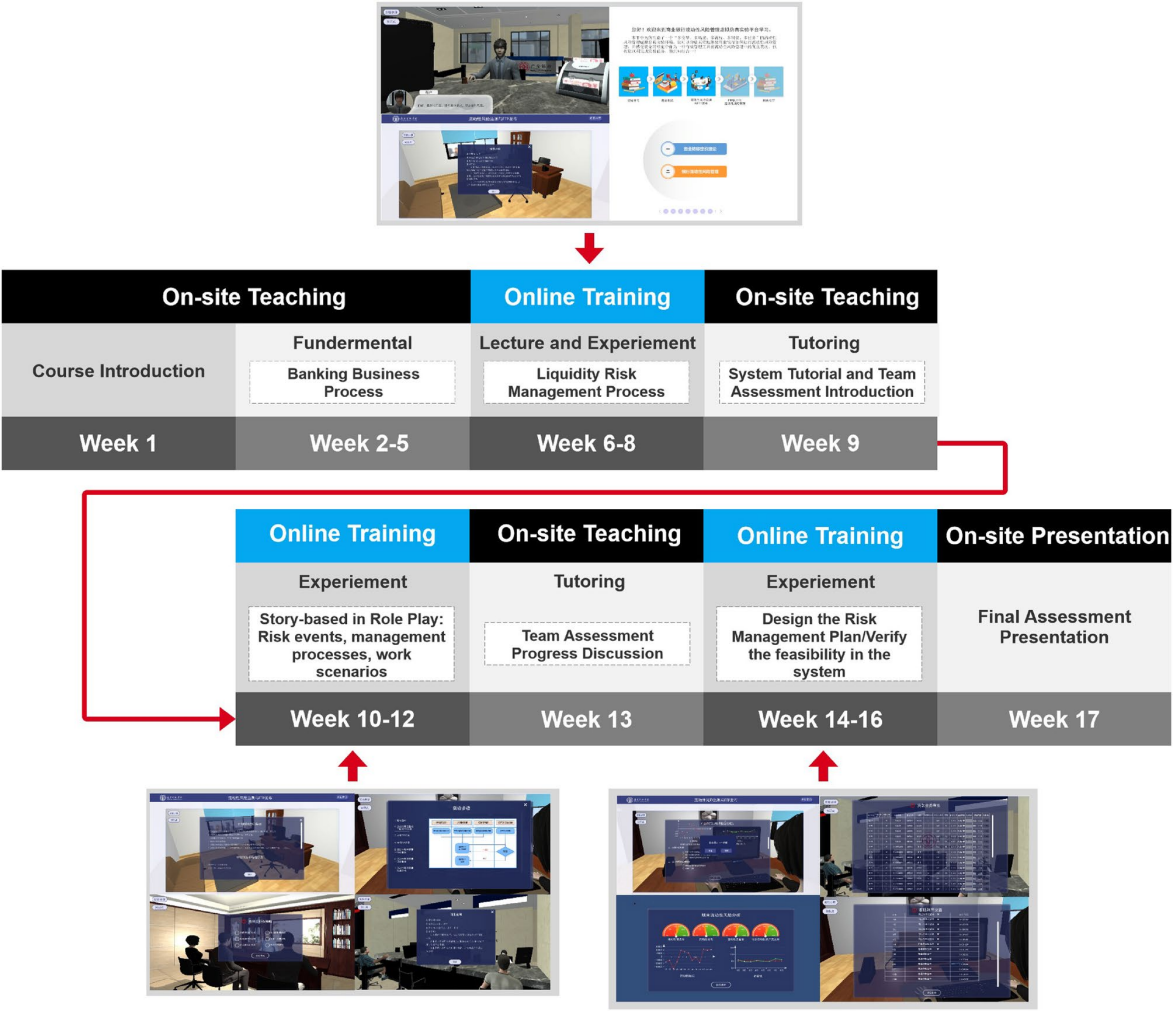
Course structure and schedule.

Figure 2 displays the course structure and schedule. The total length of the course is 17 weeks (90 minutes/week), as shown in Fig. 2. The study is divided into 7 weeks of on-site teaching, 9 weeks of online training, and 1 week for the final assessment presentation. In consideration of the potential COVID-19 pandemic, on-site teaching can be shifted to an online meeting platform based on the campus’s health conditions. The course can be separated into two sessions: fundamental lectures (weeks 1 9) and team-based assessments (weeks 10 18).

Week 1 is the course introduction, which outlines the overall strategy of the course. The teacher delivers fundamental banking business processes from week 2 to week 5, with on-site teaching. This session ensures that students have the appropriate background knowledge before entering the experimental phase. The teacher conducts online training from week 6 to week 8, with the first training session introducing the online virtual training system and subsequently using it to demonstrate the liquidity risk management process in the bank. Visualized education can help students better understand risk management progress. In week 9, the teacher begins the first on-site tutoring session to answer students’ questions about the online system and announces the team-based assessment.

During weeks 10–12, students work in teams using the training system. The teacher assigns phased tasks to help students become familiar with their roles in the system, including risk events, management processes, and work scenarios. The study aims to foster communication among team members during these tasks. The system automatically generates the phased task report after the students complete the task. In week 13, the course runs its second tutorial, during which the teacher listens to progress reports from each group and answers any questions about the final assessment. From weeks 14 to 16, each group designs and verifies a risk management proposal using the system. The team believes that the fundamental session and phased team tasks have already provided students with a solid foundation before entering the final assessment. During the final week, students present their study output in groups.

The course schedule combines the benefits of both on-site and online teaching. It provides an online training platform to help students conduct experiments and expand their career horizons. Additionally, team-based assessments can help students become familiar with teamwork as preparation for their future careers. During on-site tutoring sessions, teachers shift to a guiding role and listen to students’ voices.

## Experiment

It is necessary to measure the effectiveness of hybrid education. Therefore, the current study conducted a suitable user testing experiment. The study reviewed various existing hybrid/VT (virtual training) projects^42–44^. Demitriadou used online digital education tools to compare with traditional printed material in mathematics^42^. Joanne compared the learning efficiency between AR (augmented reality) interactive videos and handouts and found a higher learning efficiency of AR devices^43^. Based on these projects, it is important to use the comparative study method to highlight the difference between traditional and digital education. Thus, the current study used the comparative method in our user experiment.

### Participants

This study aimed to conduct a comparative analysis of the performance of various groups across different platforms, after ensuring that the underlying conditions in each group were equivalent. Thus, the study offered two fintech vocational-oriented courses in the spring semester of 2022. Table 1 illustrates detailed information. Class (Group) A followed the abovementioned hybrid education schedule. Class (Group) B followed a traditional education trajectory where students learned fintech knowledge from textbooks and lectures. However, both groups faced the same assessment and phased tasks’ submission mode and criteria. Each class had 30 students from the 2019 bachelor’s degree cohort at Guangdong University of Finance.

**Table 1:** Demographics of the participants.

	Group A	Group B
Participant number	30	30
Average age	20.3	20.1
Male:female	15:15	15:15
Language	Mandarin	Mandarin
Study background	Undergraduate student	Undergraduate student
Teaching mode	Hybrid education	Traditional education
Assessment submission	Proposal	Proposal

### Ethical approval

This study complied with all relevant guidelines and regulations. The study was conducted in accordance with the Declaration of Helsinki, and the protocol was approved by the Center of Experimental Teaching, Guangdong University of Finance. Participants signed an informed consent form, and they agreed to participate in the study.

### Study outcome data

The study aimed to measure the learning efficiency of different groups precisely. The study evaluated the student’s overall performance by analyzing the abovementioned submission materials. The study followed the project goals to measure personal learning performance. Thus, the evaluation comprised three primary aspects: ‘experimental ability’, ‘teamwork ability’, and ‘critical thinking’.

Kong concluded that experiential learning aims to enhance essential individual skills and solidify their personal foundation^45^. Additionally, Noh highlighted that self-learning can significantly enhance experiential learning in medical education^46^. Thus, the ability to experiment includes three indicators: ‘fundamental knowledge’, ‘practical ability’, and ‘self-learning’.

Kemery suggested that problem analysis and communication are weaknesses in the teamwork of undergraduate business programs^47^. Therefore, the current study organized teamwork ability into two main sections: how to analyze problems and how to communicate. In the problem-analysis section, the study emphasized students’ research output, including their writing quality and data analysis. In the communication section, the study focused on the quality of communication and how students organized teamwork and managed projects.

Spector reviewed the importance of critical thinking in developing problem-solving skills, logical reasoning, and creativity^48^. Thus, this study arranged critical thinking to consist of three indicators: ‘logic’, ‘novelty’, and ‘problem solving’.

**Figure 3:**
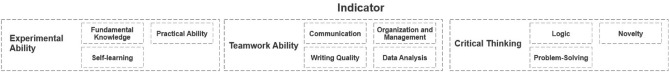
Course learning performance indicator.

Thus, there were ten indicators in total, as shown in Fig. 3. Each of these indicators was scored on a scale of 0–10. The responsibility for grading each student on these ten indicators was given to the teacher.

**Figure 4:**
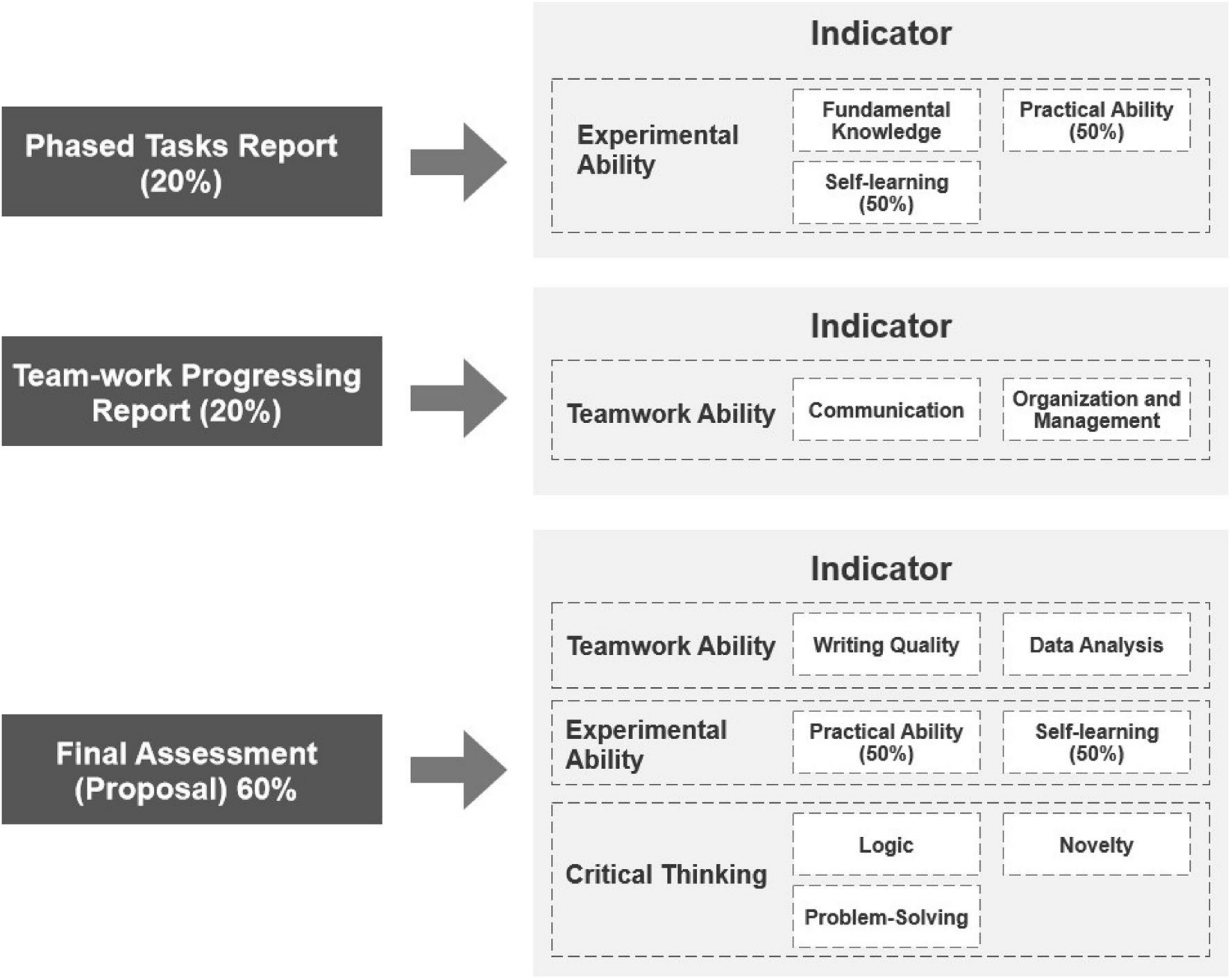
Indicator distribution.

Guangdong University of Finance requires that each experimental course places 40% of the overall weight on daily homework and 60% on the final assessment. Therefore, the study equally divided the daily homework into two parts: a phased tasks report (20%) and a teamwork progress report (20%). Both documents represented the daily individual effort. The financial risk report (proposal) served as the final assessment. Figure 4 displays the distribution of the indicators in each assessment.

A phased task report served as a fundamental practice that primarily demonstrated the students’ ‘fundamental knowledge’. Additionally, it also provided an opportunity to assess individual study progress, allowing the teacher to evaluate students’ ‘practical ability’ and ‘self-learning’. The scores for the two aspects of ‘practical ability’ and ‘self-learning’ were weighted at 50% of the indicator assessment.

The teamwork progressing report played a crucial role in assessing how team members communicated and managed their projects effectively. This report enabled the teacher to evaluate the students’ ‘communication’ and ‘organization and management’ skills based on their performance within the team. The teacher could assign scores for these two indicators based on the information provided in the teamwork progression report.

The final assessment encompassed the students’ overall study outcomes, including all three aspects. First, the teacher evaluated the overall quality of teamwork in terms of ‘written quality’ and ‘data analysis’. This assessment explained how well the team collaborated and performed in these areas. Second, the teacher monitored the individual progress of students following the phased task report. Students should continue to grow and demonstrate improvement beyond fundamental tasks. Therefore, the teacher could assign scores for ‘practical ability’ and ‘self-learning’, accounting for 50% of the indicator assessment. Last, the report reflected the progression of critical thinking skills. The teacher evaluated the logical structure of the report, looking for a coherent and well-organized presentation. Additionally, the teacher assessed whether the students’ reports demonstrated innovation (novelty) and effectively addressed the issues put forward by the students. By considering these factors in the final assessment, the teacher was able to provide a comprehensive evaluation of the students’ teamwork, individual progress, and critical thinking skills.

### Data measurement

The experiment involved not only the comparison of different groups’ performance using median and average values but also a deep analysis of the data using the entropy weight method (EWM). The EWM is an objective weighting method that determines the weight of each objective based on the variability of the corresponding index^49,50^. The information entropy of an index reflects its variation, with smaller entropy indicating greater variability^49^. Moreover, indices with higher information content have a greater impact on comprehensive evaluation and receive higher weights^49^. Equation (1) uses the maximum and minimum value standardization method to assimilate each indicator into the same range and then compare; i (i=1,2....30) represents the participants (tested students), while j (j=1,2....10) represents these evaluated aspects:

1 $$\begin{aligned} r_{ij} = \frac{X^{'}_{ij}-min\left( X^{'}_{j}\right) }{max\left( X^{'}_{j}\right) -min\left( X^{'}_{j}\right) } \end{aligned}$$

Equation (2) shows how to calculate the information entropy from a set of data:

2 $$\begin{aligned} E_{j} = -\frac{1}{\ln m}\sum _{i=1}^{m} p_{ij} \ln p_{ij} \quad p_{ij} = \frac{r_{ij}}{\sum ^{m}_{j=1} r_{ij}} \end{aligned}$$

Equation (3) determines the weight of each indicator:

3 $$\begin{aligned} w_{j} = \frac{1-E_j}{\sum ^{n}_{j=1} (1-E_{j})} \end{aligned}$$

### Data results

The study received reports from all 60 participants, achieving a completion rate of 100%. Table 2 displays the overall performance. The average summary score for Group A is 71.87, with a median score of 72. For Group B, the average summary score is 67.0, with a median score of 68. The overall data indicate that hybrid education can offer advantages over traditional education in all three aspects, with critical thinking showing the most significant improvement, having the highest increase in both average and median data.

The overall data may not reflect the students’ detailed performance; thus, the study further analyzed the data of different groups for each indicator. There are four significant trends, which are displayed in Table 3, showing the average and median scores from these aspects:

- *p values* show the significant differences between hybrid and traditional teaching in terms of ‘data analysis’, ‘communication’, ‘practical ability’, and ‘problem-solving’.
- The median data for the hybrid group suggest that their performance in ‘communication’ is 2 points higher than that of the traditional teaching group, while ‘data analysis,’ ‘novelty,’ and ‘problem-solving’ appear to show improvement with an increase of 1 point.
- The median data of the hybrid group indicates that ‘writing quality’ appears to be their weakness, with a decrease in the score of 1 point.
- The average data from the hybrid group indicates that ‘data analysis,’ ‘communication,’ ‘organization and management,’ ‘practical ability,’ ‘novelty,’ and ‘problem-solving’ all exhibit a significant improvement (more than 5%) in performance compared to in traditional teaching, with an increase of 9.76%, 14.09%, 8.11%, 9.85%, 8.57%, and 20.15%, respectively.
- The average data of the hybrid group indicate that ‘fundamental knowledge’, ‘self-learning’, and ‘logic’ all have a minor advantage (0-5%) over the same categories in traditional teaching. Furthermore, the outcome for ‘writing quality’ is lower than that in traditional teaching.

The study used EWM in Python 3.5 to analyze the further information of each indicator. To avoid calculation errors in the occurrence of zero elements of $p_{ij}$, the lowest interval of normalization can start from 0.002. Table 4 displays the entropy values and weights for the three goals. The data show that the weights differ between the two groups in ‘teamwork ability’ and ‘critical thinking’, while the finding for ‘experimental ability’ is insignificant. However, these three goals cannot provide valuable data. Therefore, the study still needed to check each indicator one by one. Table 5 displays the entropy value and weights in each group. There are four significant trends as follows:

- ‘Organization and management’, ‘fundamental knowledge’, ‘logic’, ‘self-learning’ and ‘problem-solving’ have more than 10% of the weights in Group A’s overall performance.
- ‘Practical ability’ and ‘novelty’ have less than 5% of the weights in Group A’s overall performance.
- ‘Data analysis’, ‘practical ability’, ‘self-learning’ and ‘logic’ have more than 10% of the weights in Group B’s overall performance.
- ‘Writing quality’, ‘fundamental knowledge’ and ‘novelty’ have less than 5% of the weights in Group B’s overall performance.

**Table 2:** Average and median values of the three goals and the total score.

	Median value (group A)	Average value (group A)	Median value (group B)	Average value (group B)
Experimental ability	22	21.67	21	20.67
Teamwork ability	28	28.6	27	26.73
Critical thinking	22	21.6	20	19.6
Sum	72	71.87	68	67.0

**Table 3:** Average and median values from the score distribution for each indicator.

	p-value	Median value (group A)	Average value (group A)	Median value (group B)	Average value (group B)
Writing quality	0.42	6	6.47	7	6.73
Data analysis	0.04	8	7.53	7	6.86
Communication	0.02	8	7.53	6	6.60
Organization and management	0.17	7	7.06	7	6.53
Fundamental knowledge	0.69	7	7.13	7	6.93
Practical ability	0.02	7	7.47	7	6.80
Self-learning	0.69	7	7.06	7	6.93
Logic	0.64	7	6.93	7	6.80
Novelty	0.15	7	6.73	6	6.20
Problem-solving	0.01	8	7.93	7	6.60

**Table 4:** Entropy weight method (EWM) data from the score distributions of the three goals.

	Information entropy *E* (group A)	Weights (group A) (%)	Information entropy *E* (group B)	Weights (group B) (%)
Experimental ability	0.92	45.48	0.89	40.30
Teamwork ability	0.94	30.66	0.95	20.74
Critical thinking	0.96	23.85	0.89	38.97

**Table 5:** Entropy weight method (EWM) data from the score distributions of each indicator.

	Information entropy *E* (group A)	Weights (group A) (%)	Information entropy *E* (group B)	Weights (group B) (%)
Writing quality	0.92	6.83	0.95	4.56
Data analysis	0.92	6.51	0.79	17.16
Communication	0.89	8.95	0.92	6.4
Organization and management	0.78	17.77	0.9	8.5
Fundamental knowledge	0.86	11.56	0.95	4.5
Practical ability	0.95	4.28	0.79	17.14
Self-learning	0.81	15.24	0.83	14.03
Logic	0.83	13.66	0.79	17.13
Novelty	0.94	4.44	0.95	4.29
Problem-solving	0.87	10.75	0.92	6.32

### Data analysis

This section starts by analyzing the average and median data between the two groups. The EWM weight data aim to help us deeply analyze the advantages/disadvantages of indicators in the overall score distribution. The current study aimed to not only find the connection between the advantages but also to identify the relationship between weak indicators and insignificant advantages.

The abovementioned data demonstrate the significant advantages of hybrid education in several indicators. The virtual training system successfully assists students in interacting with data and helps them build a solid knowledge structure in data analysis. Online cooperation also helps to build team relationships and leads to good organization. Furthermore, the experimental session significantly improves their practical abilities, while the immersive environment can motivate them to continue learning beyond the classroom. Students can be attracted to broaden their horizons in self-learning and improve their innovative abilities. Group A’s EWM weight data also support that teamwork organization and self-learning occupy a significant position in the overall scores.

Apart from the significant advantages, there are some minor advantages to ‘fundamental knowledge’ and ‘novelty’ in other indicators. In addition, the study also identified a weakness in ‘writing quality’. The average and median data indicate that Group A’s performance in ‘writing quality’ is inferior to that of Group B’s. The EWM data also show that ‘writing quality’ carries a weight of only 6.83% in the overall score.

## Discussion

The user data underscore a positive outcome in hybrid education with an immersive experience. Notably, VR technology has shown remarkable advantages, particularly in improving students’ communication skills. This aligns with VR’s potential to play an innovative role in collaborative work in Industry 4.0^51^.

Concurrently, VR training modules bolster students’ foundational knowledge and practical skills, providing them with insights into authentic working environments, which is a dimension that is typically unavailable through traditional on-site study. This exposure equips students with a more profound understanding of industry-specific standards. The positive results from the experimental study on virtual training are consistent with the advantages of VR in this domain, as outlined by^52^.

Throughout the immersive learning process, students have the opportunity to expand their intellectual horizons and foster creative thinking. The training modules also aid in developing a logical approach to problem solving, aligning with recent research that suggests VR learning can enhance participants’ critical thinking skills^53^.

However, the data still reveal certain weaknesses in writing skills. The traditional education mode dedicates a substantial portion of the 16-week teaching period to lectures that introduce a variety of fintech projects and articles. This extended timeframe allows students to acquire a comprehensive understanding of diverse definitions and other related concepts in the classroom. In contrast, the hybrid mode emphasizes experiments, with the training system lacking specific writing skill training content. The fewer provided lectures (8 weeks) in Group A result in limited advantages in ‘fundamental knowledge’ and expose a weakness in ‘writing quality’. Additionally, the VR system concentrates on data visualization and analysis, thereby enabling the generation of reports but not providing ample practice for writing.

Taking the abovementioned data into consideration, it is essential to strike a balance in the ratio between lectures and practical experiments. Therefore, future studies should persist in refining the course structure and enhancing the efficiency of knowledge acquisition during lectures. The advent of artificial intelligence (AI) languages offers the potential to assist students as virtual teaching assistants^54^. In this regard, the VR system should incorporate additional AI-assisted modules to support students further. These improvements have the potential to enhance lecture comprehension and writing quality during the final assessment.

## Conclusion

The COVID-19 pandemic has exacerbated adverse effects on the fintech employment market in China, with high barriers to entry based on profitability, risk, security, and confidentiality limiting students’ access to the industry. Therefore, it is essential to help students transition to their careers before graduation. Given the advantages of VR technology, the current study used a hybrid mode to improve a fintech course, addressing existing challenges during the pandemic with a conceptual framework based on OBE theory and the flipped classroom model. The study designed a detailed course structure with on-site teaching/tutorials and online experiments supported by an online VR training platform. A comparative user experiment with 60 participants demonstrated that hybrid education positively impacts individual critical thinking, communication, and teamwork skills, consistent with prior research^51–53,55–57^. However, the current study also revealed the limitations of hybrid education, particularly in terms of individual writing skills and fundamental knowledge. These findings suggest that the hybrid course structure and teaching methods need to be continually refined to optimize the benefits of hybrid education. Further research could investigate the most effective ways to combine online (VR) and AI-assisted learning to achieve the best possible outcomes in fintech career education. Overall, the current study provides critical data references for global online/hybrid education researchers and contributes to the education field.

Although this study has made significant contributions to the field of hybrid education, there are still some limitations that need to be addressed. As the reviewer suggested, we have carefully revised the conclusion section to highlight the data results and possible steps for future work. Additionally, as the sample size of the study is relatively small, it is recommended that the experimental group be expanded in the future to obtain more stable EWM data and provide more critical data analysis. In addition, further studies should investigate the long-term impact of hybrid education on student learning outcomes and evaluate the effectiveness of various hybrid course designs. In contrast, the current hybrid study took place over the course of only one semester. Our future research should focus on investigating the long-term impact of hybrid education on student learning outcomes. By conducting longitudinal studies, researchers can gain a deeper understanding of how hybrid education influences students’ academic performance, skill development, and overall learning experience over an extended period.

## Data Availability

The datasets used and analyzed during the current study are available from the corresponding author upon reasonable request.
